# Dataset of Batik Nitik Sarimbit 120

**DOI:** 10.1016/j.dib.2024.110671

**Published:** 2024-06-25

**Authors:** Agus Eko Minarno, Indah Soesanti, Hanung Adi Nugroho

**Affiliations:** a Department of Electrical and Information Engineering, Universitas Gadjah Mada, Indonesia; b Department of Information Technology, Universitas Muhammadiyah Malang, Indonesia

**Keywords:** Batik, Nitik, Art, Heritage, Generative adversarial network, Diffusion, Autoencoder

## Abstract

Batik holds profound cultural significance within Indonesia, serving as a tangible expression of the nation's rich heritage and intricate philosophical narratives. This paper introduces the Batik Nitik Sarimbit 120 dataset, originating from Yogyakarta, Indonesia, as a pivotal resource for researchers and enthusiasts alike. Comprising images of 60 Nitik patterns meticulously sourced from fabric samples, this dataset represents a curated selection of batik motifs emblematic of the region's artistic tradition. The Batik Nitik Sarimbit 120 dataset offers a comprehensive collection of 120 motif pairs distributed across 60 distinct categories. By providing a comprehensive repository of batik motifs, the Batik Nitik Sarimbit 120 dataset facilitates the training and validation of machine learning algorithms, particularly through the utilization of generative method. This enables researchers to explore and innovate in the realm of batik pattern generation, fostering new avenues for creativity and expression within this venerable art form. In essence, the Batik Nitik Sarimbit 120 dataset stands as a testament to the collaborative efforts of cultural institutions and academia in preserving and promoting Indonesia's rich batik heritage. Its accessibility and richness make it a valuable resource for scholars, artists, and enthusiasts seeking to delve deeper into the intricate world of Indonesian batik.

Specifications Table

**Table:** 

Subject	Computer Science
Specific subject area	Computer Science Applications, Computer Vision and Pattern Recognition
Type of data	Image
Data collection	Data collection utilized a Sony Alpha 6400 camera equipped with an APS-C sensor, featuring a resolution of 24MP and image dimensions of 6024×4024 pixels. Double lighting was employed utilizing the Godox SKII400, triggered by the Godox X2T for Sony. The lens used was the Sony 80–135 mm, capturing original images in JPG format and RGB color.
Data source location	• Institution: Paguyuban Pecinta Batik Indonesia (PPBI) Sekar Jagad • City/Town/Region: Yogyakarta • Country: Indonesia
Data accessibility	All the images have been uploaded to an open, free-to-use research data repository named “Mendeley Data.” The specific details to access the images data are: Minarno, Agus Eko; Soesanti, Indah; Nugroho, Hanung Adi (2024), “Batik Nitik Sarimbit 120″, Mendeley Data, V2, doi: 10.17632/cx5sr2dgrr.2 Repository name: Mendeley Data Data identification number: 10.17632/cx5sr2dgrr.2 Direct URL to data: http://dx.doi.org/10.17632/cx5sr2dgrr.2
Related research article	

## 1 Value of the Data

• Batik Nitik Sarimbit 120 was built using existing original batik cloth and not using vector images.
• The dataset consists of 60 categories of batik motifs and each category has a pair so the total is 120 motifs.Motif sets provide utility in computer vision and pattern recognition contexts, enabling functions such as those found in Generative Adversarial Network (GAN), Diffusion, or Autoencoder operations.
• Batik Nitik Sarimbit 120 is useful for innovating new methodologies in producing new batik designs by utilizing GAN, Diffusion or Autoencoder operations.
• Each Batik Nitik Sarimbit 120 image has a counterpart, making it easier for the training stage and allowing it to be combined with other motifs, so that new motifs can be formed.

## 2 Background

Batik Nitik embodies a valuable artistic creation with significant economic implications; however, the availability of its designs is notably restricted, thereby necessitating preservation efforts facilitated by Artificial Intelligence (AI) technologies capable of engendering novel designs. Through the introduction of fresh design ideas, batik artisans can produce a broader array of works, thereby fostering economic growth. Additionally, batik has emerged as a subject of research within the field of computer science, particularly concerning classification, retrieval, and generative adversarial network techniques. Regrettably, the current accessibility of public batik datasets remains severely limited [1]. By disseminating batik data, it is anticipated that research endeavors concerning batik will proliferate, thus benefiting researchers, while the outcomes of such research may be leveraged by batik artisans to bolster economic prosperity. Batik, a cultural heritage of significant importance, encompasses two primary pattern categories: geometric and non-geometric. Indonesia boasts over a thousand distinct batik patterns, each presenting challenges in terms of recognition and differentiation. Concurrently, there is a demand for innovative approaches to enhance the diversity of batik motifs, thus facilitating the creation of new designs. To address these challenges, various research efforts focusing on generating new motif using generative adversarial networks [[2], [3], [4], [5], [6]] and diffusion [7] have been initiated. Although the Batik Nitik 960 dataset, introduced by Minarno [8], is valuable, it lacks comprehensive identification motif pairs. This dataset presents batik images along with details and motif pairs [9]. Notably, Batik Nitik Sarimbit 120 emerges as the primary publicly accessible dataset featuring comprehensive including motif names and motif pairs.

The Batik Nitik Sarimbit 120 dataset is designed with paired motifs for each pattern, making it an ideal resource for applications in Generative Adversarial Networks, diffusion models, and Autoencoders. In the context of GAN, the dataset can be used to train the generator to create new Batik motifs by learning the distribution and characteristics of the existing patterns. The paired nature of the dataset allows the GAN to better understand the relationship between different motifs, thereby generating more coherent and authentic designs. For diffusion models, the dataset provides a basis for modeling the gradual process of pattern evolution. By using the pairs, diffusion models can be trained to predict the transformation from one motif to its corresponding pair, capturing the intricate changes in pattern details. This can lead to the creation of smoother and more natural-looking Batik motifs. In the case of Autoencoders, the paired motifs in the dataset enable the encoder-decoder architecture to learn efficient representations of the Batik patterns. The encoder compresses the motif information into a latent space, while the decoder reconstructs the patterns from this latent representation. The pairs help in training the Autoencoder to recognize and reproduce the subtle variations between different motifs, enhancing its ability to generate high-quality Batik designs.

## 3 Data Description

The Batik Nitik motif, a distinguished pattern with its origins traced back to Yogyakarta, stands as a testament to the rich cultural heritage of the region. This intricate motif was meticulously crafted by artisans associated with the Yogyakarta Palace, notably by BRAy. Brongtodiningrat, marking its inception on February 19, 1940. Initially, the creative endeavors of BRAy. Brongtodiningrat led to the development of 56 distinct Nitik motifs. However, the evolution of this artistic tradition did not cease there. Subsequently, Hani Winotosastro made significant contributions to the Nitik motif collection. By adding four additional motifs, Hani's efforts culminated in a comprehensive array of 60 motifs. Among these additions were the enchanting Sekar Jeruk, Sekar Srengenge, Sekar Sawo, and Sekar Gambir motifs. Through Hani's meticulous craftsmanship and creative vision, the Nitik motif collection achieved a remarkable level of completeness and diversity.

**Fig. 1:**
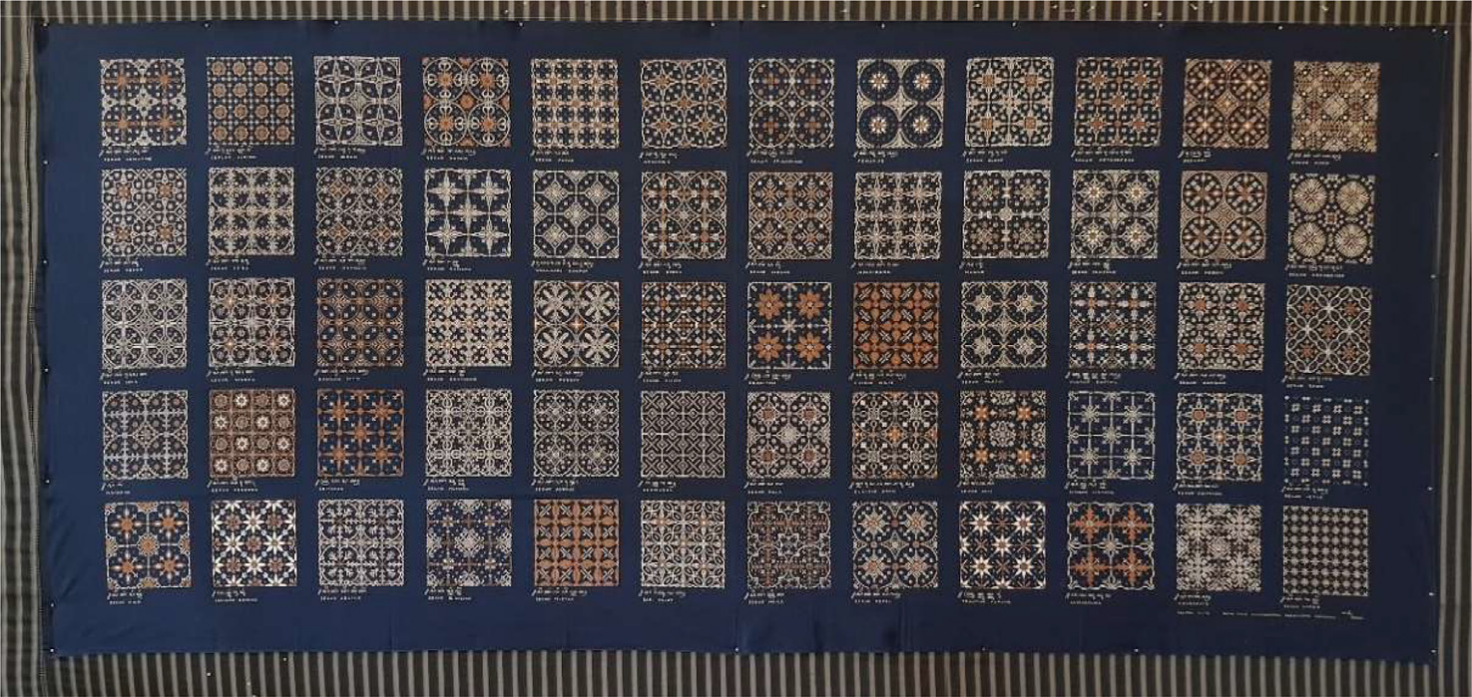
In 2010, a remastered version of the Batik Nitik fabric sample owned by Winotosastro was produced.

The significance of this achievement is further underscored by the visual representation of the Nitik Batik fabric, which elegantly showcases all 60 motifs attributed to Hani Winotosastro, as depicted in Fig. 1. This representation not only serves as a visual testament to the intricate beauty of the Nitik motifs but also embodies the dedication and artistry of the individuals involved in preserving and advancing the tradition of Batik Nitik.

**Fig. 2:**
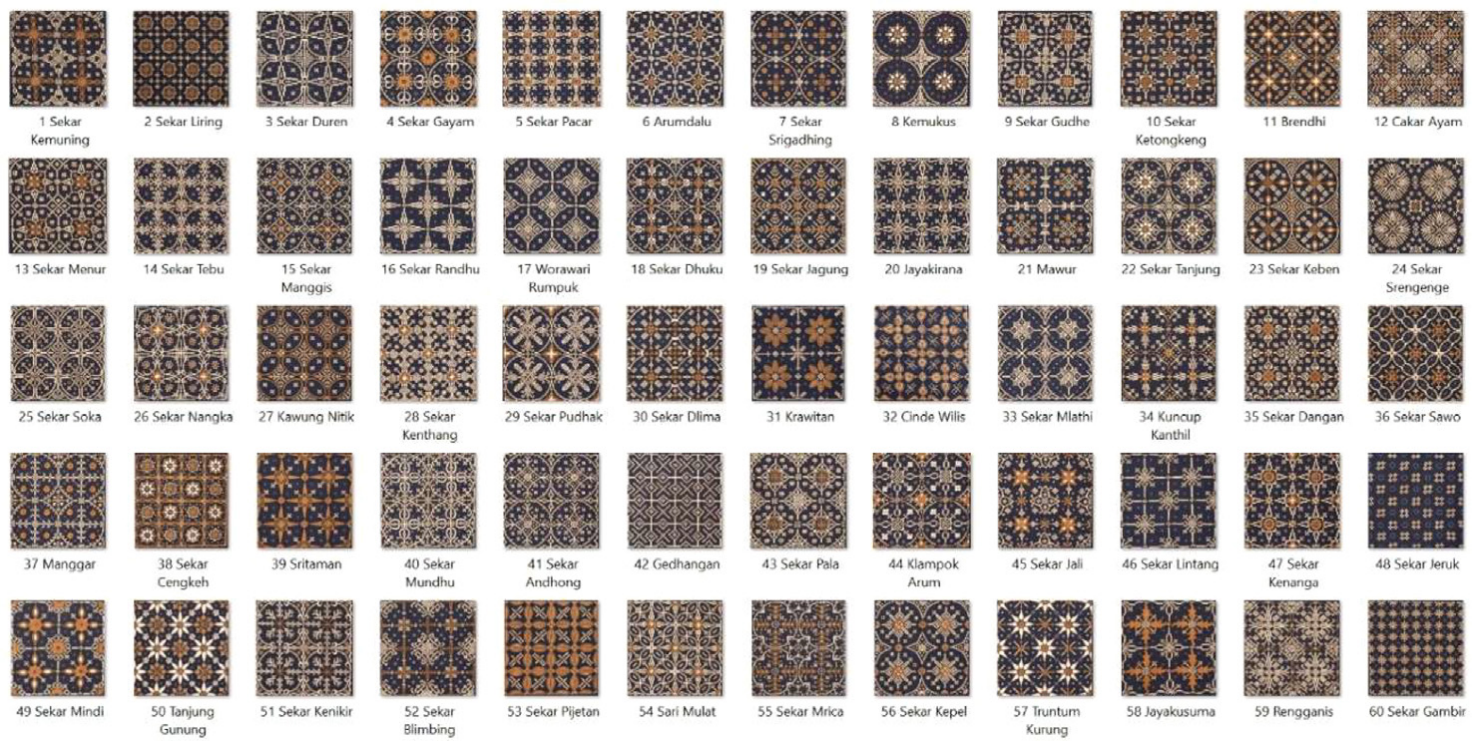
The 60 motif of Batik Nitik.

The dataset is organized into 60 categories, encompassing a total of 120 images. Each image is formatted in jpg with dimensions of 512×512 pixels. The original 60 categories, each detailed with the name of the motif, are illustrated in Fig. 2. The sample of specifics of these categories are outlined in Table 1.

**Table 1:** The sample of motif names and pairs of Batik Nitik Sarimbit 120.

## 4 Experimental Design, Materials and Methods

**Fig. 3:**
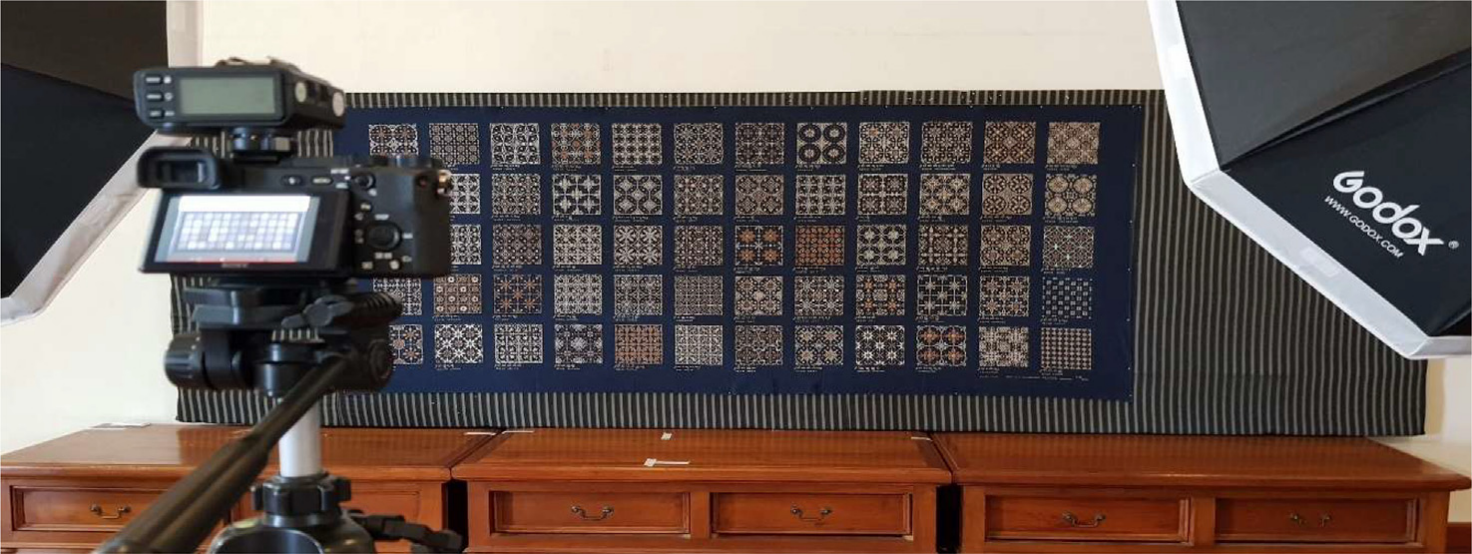
Process capturing Batik Nitik Sarimbit 120.

The photographs were taken in September 2022 at APIP's Batik studio in Yogyakarta, Indonesia, which measures 10×8 m. The Batik Nitik fabric was captured using a Sony Alpha 6400 camera with a 2-set Godox II SK 400 lighting setup and a Sony lens of 85–135 mm. The camera's settings were adjusted to an aperture of F1/10, a shutter speed of 1/10, ISO 200, a focal length of 135 mm, a white balance set to 5500 K, and a flash intensity of 1/16. Images were captured with dimensions of 6024×4024 pixels in jpg format.. Each Nitik motif was individually photographed to capture detailed textures. Fig. 3 illustrates the process of capturing the Batik Nitik fabric. The image pre-processing involved three key phases:

1. Image Cropping
  The motifs, originally captured in jpg format, were individually cropped out manually, requiring 60 separate cropping operations to isolate each piece. This meticulous cropping process is depicted in Fig. 4.

**Fig. 4:**
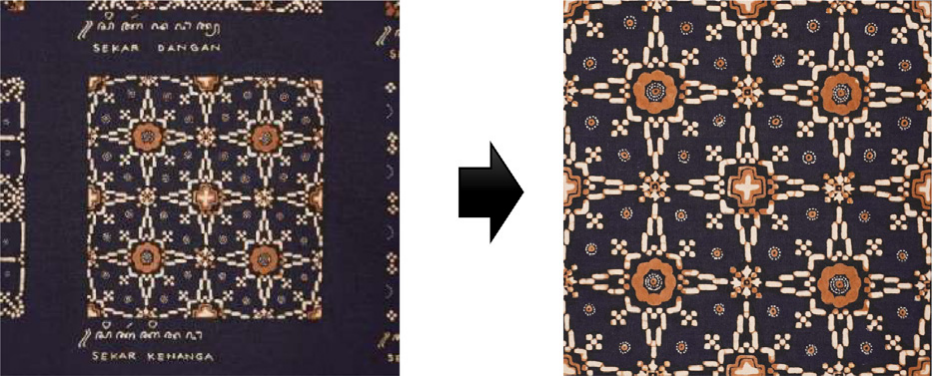
Pre-processing stage; cropping each sample piece Nitik.
2. Image Splitting
  The next step involved dividing sample motifs into two separate images. The method for this division is showcased in Fig. 5.

**Fig. 5:**
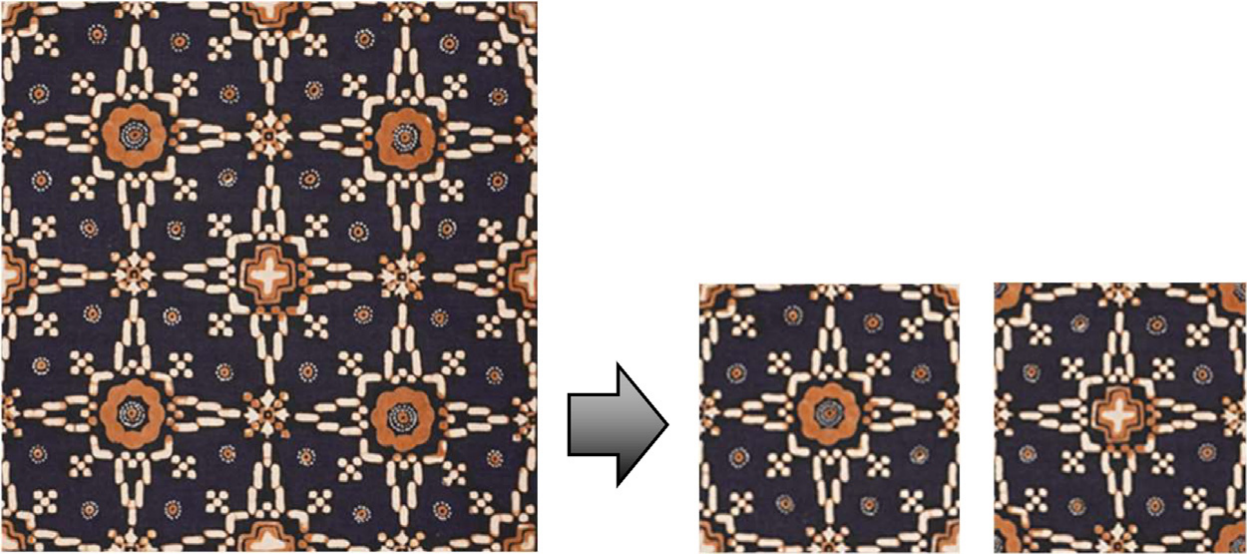
Pre-processing stage; splitting each sample piece Nitik motif into two pieces motif.
3. The final dataset is presented in Fig. 6**.**

**Fig. 6:**
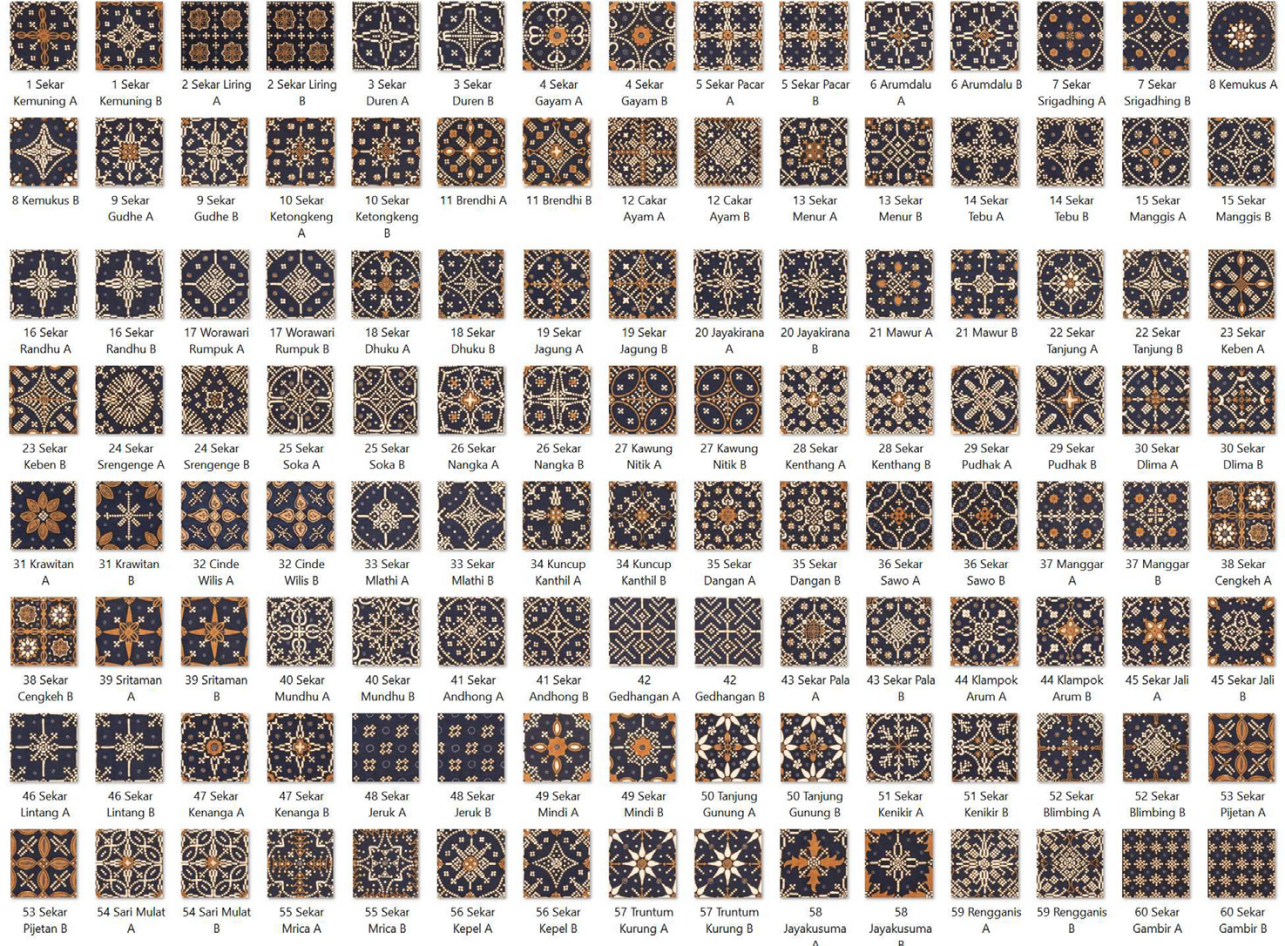
The final result of Batik Nitik Sarimbit 120.

## Limitations

Not applicable.

## Ethics Statement

The authors declare that there are no ethical issues with the data presented.

## CRediT authorship contribution statement

**Agus Eko Minarno:** Writing – review & editing, Methodology, Conceptualization. **Indah Soesanti:** Writing – review & editing, Investigation. **Hanung Adi Nugroho:** Writing – review & editing.

## Data Availability

Batik Nitik Sarimbit 120 (Original data) (Mendeley Data).
